# Supplementary material for: Low Frequency of Cancer-Predisposition Gene Mutations in Liver Transplant Candidates with Hepatocellular Carcinoma
Source: Cancers (Basel). 2022 Dec 29;15(1):201. doi: 10.3390/cancers15010201 (PMC9818325; doi:10.3390/cancers15010201)
Supplement: Supplementary file 1 [file cancers-15-00201-s001.zip › cancers-2100821-supplementary.pdf]

Low frequency of cancer-predisposition gene mutations in liver transplant candidates with hepatocellular carcinoma

Klara Horackova, Sona Frankova, Petra Zemankova, Petr Nehasil, Marta Cerna, Magdalena Neroldova, Barbora Otahalova, Jan Kral, Milena Hovhannisyan, Viktor Stranecky, Tomas Zima, Marketa Safarikova, Marta Kalousova, CZECANCA consortium, Jan Novotny, Jan Sperl, Marianna Borecka, Sandra Jelinkova, Michal Vocka, Marketa Janatova, Petra Kleiblova, Zdenek Kleibl, Milan Jirsa, Jana Soukupova

SUPPLEMENTARY MATERIALS – TABLE OF CONTENTS:

**Table S1: List of CZECANCA consortium members**
**Table S2: List of 226 genes included in the CZECANCA panel (and used transcription variants), divided into two groups based on their clinical significance.**
**Table S3: Clinicopathological characteristics in 334 HCC patients' subgroups of carriers PV in cancer-predisposition genes (any CPG) and non-carriers (None).**
**Table S4: Characterization of HCC patients from retrospective CZECANCA consortium database.**
**Figure S1: Survival of variant carriers in A. established genes, B. candidate genes, C. MRN genes compared to non-carriers.**

Table S1: List of CZECANCA consortium members

Institution	Members
Institute of Biochemistry and Experimental Oncology, First Faculty of Medicine, Charles University, Prague, Czech Republic	Zdenek Kleibl, Jana Soukupova, Marketa Janatova Petra Zemankova, Marianna Borecka
Department of Cancer Epidemiology and Genetics, Masaryk Memorial Cancer Institute, Brno, Czech Republic	Eva Machackova, Lenka Foretova, Dita Hanouskova
Department of Medical Genetics, AGEL Laboratories, AGEL Research and Training Institute, Novy Jicin, Czech Republic	Spiros Tavandzis, Vera Krutilkova, Barbora Roszkova
Centre for Medical Genetics and Reproductive Medicine, Gennet, Prague, Czech Republic	Monika Koudova, Stepan Chvojka, Leona Cerna Jana Vavrova
Institute of Biology and Medical Genetics, General University Hospital and First Faculty of Medicine, Charles University, Prague, Czech Republic	Petra Kleiblova, Michal Vocka, Jan Novotny Ondrej Havranek
Department of Medical Genetics, GHC Genetics, Prague, Czech Republic	Lucie Hruskova, Renata Michalovska, Zdenka Vlckova Denisa Schwetzova
Institute of Medical Genetics, University Hospital Pilsen, Pilsen, Czech Republic.	Monika Cerna, Marketa Hejnalova, Nikol Jedlickova Ivan Subrt, Tomas Zavoral
Department of Medical Genetics, Pronatal, Prague, Czech Republic	Marcela Kosarova, Klara Kozakova, Alena Fullsackova Gabriela Vacinova
Department of Medical Genetics, University Hospital Olomouc, Faculty of Medicine and Dentistry, Palacky University Olomouc, Czech Republic	Maria Janikova, Romana Kratochvilova, Vaclava Curtisova
Laboratory of Molecular Genetics, Hospital Ceske Budejovice, Ceske Budejovice, Czech Republic.	Ondrej Scheinost, Petra Duskova
Department of Paediatrics and Inherited Metabolic Disorders, First Faculty of Medicine, Charles University and General University Hospital in Prague, Czech Republic.	Viktor Stranecky, Petr Nehasil
Laboratory of Cancer Cell Biology, Institute of Molecular Genetics of the Czech Academy of Sciences, Prague, Czech Republic.	Libor Macurek

Table S2: List of 226 genes included in the CZECANCA panel (and used transcription variants), divided into two groups based on their clinical significance.

**Established high-to-moderate CPG** (N = 48)	***APC*** (NM_000038), ***ATM*** (NM_000051), ***BAP1*** (NM_004656), ***BARD1*** (NM_000465), ***BMPR1A*** (NM_004329), ***BRCA1*** (NM_007294), ***BRCA2*** (NM_000059), ***BRIP1*** (NM_032043), ***CDH1*** (NM_004360), ***CDK4*** (NM_000075), ***CDKN2A*** (NM_000077), ***CHEK2*** (NM_007194), ***EPCAM*** (NM_002354), ***FH*** (NM_000143), ***FLCN*** (NM_144997), ***KIT*** (NM_000222), ***MEN1*** (NM_000244), ***MET*** (NM_001127500), ***MLH1*** (NM_000249), ***MSH2*** (NM_000251), ***MSH6*** (NM_000179), ***MUTYH**** (NM_001128425), ***NBN*** (NM_002485), ***NF1*** (NM_000267), ***NF2*** (NM_000268), ***PALB2*** (NM_024675), ***PMS2*** (NM_000535), ***POLD1*** (NM_002691), ***POLE*** (NM_006231), ***PRKAR1A*** (NM_212471), ***PTEN*** (NM_000314), ***PTCH1*** (NM_000264), ***RAD51C*** (NM_058216), ***RAD51D*** (NM_002878), ***RB1*** (NM_000321), ***RET*** (NM_020975), ***SDHB*** (NM_003000), ***SDHC*** (NM_003001), ***SDHD*** (NM_003002), ***SMAD4*** (NM_005359), ***SMARCB1*** (NM_003073), ***STK11*** (NM_000455), ***SUFU*** (NM_016169), ***TP53*** (NM_000546), ***TSC1*** (NM_000368), ***TSC2*** (NM_000548), ***VHL*** (NM_000551), ***WT1*** (NM_024426)
**Candidate CPG** (N = 178)	***AIP*** (NM_003977), ***ALK*** (NM_004304), ***APEX1*** (NM_001641), ***ATMIN*** (NM_015251), ***ATR*** (NM_001184), ***ATRIP*** (NM_130384), ***AURKA*** (NM_198433), ***AXIN1*** (NM_003502), ***BABAM1*** (NM_001033549), ***BLM*** (NM_000057), ***BRAP*** (NM_006768), ***BRCC3*** (NM_024332), ***BRE*** (NM_004899), ***BUB1B*** (NM_001211), ***CASP8*** (NM_001080125), ***CCND1*** (NM_053056), ***CDC73*** (NM_024529), ***CDKN1B*** (NM_004064), ***CDKN1C*** (NM_000076), ***CEBPA*** (NM_004364), ***CEP57*** (NM_014679), ***CHEK1*** (NM_001114122), ***CLSPN*** (NM_022111), ***CSNK1D*** (NM_001893), ***CSNK1E*** (NM_001894), ***CWF19L2*** (NM_152434), ***CYLD*** (NM_015247), ***DCLRE1C*** (NM_001033855), ***DDB2*** (NM_000107), ***DHFR*** (NM_000791), ***DICER1*** (NM_030621), ***DIS3L2*** (NM_152383), ***DMBT1*** (NM_007329), ***DMC1*** (NM_007068), ***DNAJC21*** (NM_194283), ***DPYD*** (NM_000110), ***EGFR*** (NM_005228), ***EMSY*** (NM_001300942), ***EPHX1*** (NM_000120), ***ERCC1*** (NM_001983), ***ERCC2*** (NM_000400), ***ERCC3*** (NM_000122), ***ERCC4*** (NM_005236), ***ERCC5*** (NM_000123), ***ERCC6*** (NM_000124), ***ESR1*** (NM_000125), ***ESR2*** (NM_001437), ***EXO1*** (NM_006027), ***EXT1*** (NM_000127), ***EXT2*** (NM_000401), ***EYA2*** (NM_005244), ***EZH2*** (NM_004456), ***FAAP24*** (NM_152266), ***FAM175A*** (NM_139076), ***FAM175B*** (NM_032182), ***FAN1*** (NM_014967), ***FANCA*** (NM_000135), ***FANCB*** (NM_001018113), ***FANCC*** (NM_000136), ***FANCD2*** (NM_033084), ***FANCE*** (NM_021922), ***FANCF*** (NM_022725), ***FANCG*** (NM_004629), ***FANCI*** (NM_001113378), ***FANCL*** (NM_001114636), ***FANCM*** (NM_020937), ***FBXW7*** (NM_033632), ***GADD45A*** (NM_001924), ***GATA2*** (NM_001145661), ***GPC3*** (NM_004484), ***GRB7*** (NM_001242442), ***HELQ*** (NM_133636), ***HNF1A*** (NM_001306179), ***HOXB13*** (NM_006361), ***HRAS*** (NM_005343), ***HUS1*** (NM_004507), ***KAT5*** (NM_182710), ***KCNJ5*** (NM_000890), ***LIG1*** (NM_000234), ***LIG3*** (NM_013975), ***LIG4*** (NM_002312), ***LMO1*** (NM_002315), ***LRIG1*** (NM_015541), ***MAX*** (NM_002382), ***MCPH1*** (NM_024596), ***MDC1*** (NM_014641), ***MDM2*** (NM_002392), ***MDM4*** (NM_002393), ***MGMT*** (NM_002412), ***MLH3*** (NM_001040108), ***MMP8*** (NM_002424), ***MPL*** (NM_005373), ***MRE11A*** (NM_005591), ***MSH3*** (NM_002439), ***MSH5*** (NM_002441), ***MSR1*** (NM_138715), ***MUS81*** (NM_025128), ***NAT1*** (NM_000662), ***NCAM1*** (NM_001242607), ***NELFB*** (NM_015456), ***NFKBIZ*** (NM_031419), ***NHEJ1*** (NM_024782), ***NSD1*** (NM_022455), ***OGG1*** (NM_002542), ***PARP1*** (NM_001618), ***PCNA*** (NM_002592), ***PHB*** (NM_002634), ***PHOX2B*** (NM_003924), ***PIK3CG*** (NM_002649), ***PLA2G2A*** (NM_000300), ***PMS1*** (NM_000534), ***POLB*** (NM_002690), ***PPM1D*** (NM_003620), ***PREX2*** (NM_024870), ***PRF1*** (NM_001083116), ***PRKDC*** (NM_006904), ***PTTG2*** (NM_006607), ***RAD1*** (NM_002853), ***RAD17*** (NM_133338), ***RAD18*** (NM_020165), ***RAD23B*** (NM_002874), ***RAD50*** (NM_005732), ***RAD51*** (NM_133487), ***RAD51AP1*** (NM_001130862), ***RAD51B*** (NM_133509), ***RAD52*** (NM_134424), ***RAD54B*** (NM_012415), ***RAD54L*** (NM_003579), ***RAD9A*** (NM_004584), ***RBBP8*** (NM_002894), ***RECQL*** (NM_002907), ***RECQL4*** (NM_004260), ***RECQL5*** (NM_004259), ***RFC1*** (NM_002913), ***RFC2*** (NM_181471), ***RFC4*** (NM_181573), ***RHBDF2*** (NM_024599), ***RNF146*** (NM_001242844), ***RNF168*** (NM_152617), ***RNF8*** (NM_003958), ***RPA1*** (NM_002945), ***RUNX1*** (NM_001754), ***SBDS*** (NM_016038), ***SDHA*** (NM_004168), ***SDHAF2*** (NM_017841), ***SETBP1*** (NM_015559), ***SETX*** (NM_015046), ***SHPRH*** (NM_001042683), ***SLX4*** (NM_032444), ***SMARCA4*** (NM_001128849), ***SMARCE1*** (NM_003079), ***TCL1A*** (NM_021966), ***TELO2*** (NM_016111), ***TERF2*** (NM_005652), ***TERT*** (NM_198253), ***TLR2*** (NM_003264), ***TLR4*** (NM_138554), ***TMEM127*** (NM_017849), ***TOPBP1*** (NM_007027), ***TP53BP1*** (NM_001141980), ***TSHR*** (NM_000369), ***UBE2A*** (NM_003336), ***UBE2B*** (NM_003337), ***UBE2I*** (NM_003345), ***UBE2V2*** (NM_003350), ***UBE4B*** (NM_001105562), ***UIMC1*** (NM_001199297), ***WRN*** (NM_000553), ***XPA*** (NM_000380), ***XPC*** (NM_004628), ***XRCC1*** (NM_006297), ***XRCC2*** (NM_005431), ***XRCC3*** (NM_005432), ***XRCC4*** (NM_022406), ***XRCC5*** (NM_021141), ***XRCC6*** (NM_001469), ***ZNF350*** (NM_021632), ***ZNF365*** (NM_014951)

* only in case of homozygote or compound heterozygote.

Table S3: Clinicopathological characteristics in 334 HCC patients' subgroups of carriers PV in cancer-predisposition genes (any CPG) and non-carriers (None). The characteristics in all patients and in PV carrier subgroups did not differ from that in non-carriers except for those denoted by an asterisk (* <0.05).

	**Any CPG** N=47	**Established CPG (N=7)**	**Candidate CPG (N=40)**	**None** N=287
**Age** [years]; median (range)	65 (40-77)	69 (53-72)	64 (40-77)	63 (26-76)
**Cirrhosis**; N= (%)	**45 (95.7)**	**7 (100)**	**38 (95)**	**284 (99)**
Alcoholic	12 (25.5)	3 (42.9)	9 (22.5)	117 (40.8)
Viral	18 (38.3)	3 (42.9)	15 (37.5)	102 (35.5)
Cholestatic & autoimmune	10 (21.3)	1 (14.3)	9 (22.5)	38 (13.2)
NASH (non-alcoholic steatohepatitis)	5 (10.6)	0	5 (12.5)	24 (8.4)
Metabolic	0	0	0	3 (1.0)
**None**	**2 (4.3)**	**0**	**2 (5.0)**	**3 (1.0)**
**Treatment**; N= (%)				
Liver transplantation	42 (89.4)	5 (71.4)	37 (92.5)	257 (89.5)
Other	5 (10.6)	2 (28.6)	3 (7.5)	30 (10.4)
**HCC characteristics**				
AFP [ng/mL]; median (range)	8 (2-1210)	9.6 (2-1210)	6.5 (2-699)	
Milan criteria; N= (% of known)	30 (63.8)	4 (57.1)	26 (65.0)	190 (66.2)
Microangioinvasion; N= (% of known)	21 (55.3)	3 (60.0)	18 (54.5)	107 (44.0)
Cholangiocarc. differentiation; N= (% of known)	0	0	0	18 (7.1)
**Grading**; N= (% of known)				
1	5 (12.5)	1 (16.7)	4 (11.8)	32 (13.1)
2	19 (47.5)	2 (33.3)	17 (50.0)	135 (55.1)
3	16 (40.0)	3 (50.0)	13 (38.2)	78 (31.8)
**Second primary tumor**; N= (%)	10 (21.4)	3 (42.9)	7 (17.5)	47 (16.4)
**Malignancy in 1st/2nd degree relatives**; N= (%)	22 (46.8)	3 (42.9)	19 (47.5)	109 (38.0)
**Diabetes**; N= (%)	16 (34.0)	1 (14.3)	15 (37.5)	122 (42.5)
**Obesity (BMI>30)**; N= (%)	9 (19.1)	2 (28.6)	7 (17.5)	85 (29.6)
**Smoking**; N= (%)	19 (40.4)	2 (28.6)	17 (42.5)*	173 (60.3)*

Table S4: Characterization of HCC patients from retrospective CZECANCA consortium database. Positive cancer-related characteristics are highlighted in bold.

Pts #	Mutation	Age at HCC	Other tumor	Family cancer history	
**CZE_1**	0	39	0	**positive**	EC (mother@46), RCC (father@58), BC (mother's sister)
**CZE_2**	***BRCA1*: c.1016delA (p.Lys339fs)**	44	0	**positive**	OvC (mother@36), RCC (mother's sister)
**CZE_3**	0	70	0	**positive**	GaC (father, sister)
**CZE_4**	0	NA	0	**positive**	PrC (mother's father@58), OvC (mother's mother@55)
**CZE_5**	0	NA	**BC, RCC**	negative	0
**CZE_6**	0	NA	**RCC**	negative	0
**CZE_7**	***CHEK2*: c.1100delC (p.Thr367fs)**	NA	**Bilateral BC**	**positive**	PrC (father)
**CZE_8**	0	NA	**EC, LuC**	negative	0
**CZE_9**	0	33	0	**positive**	BC (mother's mother, mother's sister 1@56, mother's sister 2@50), PaC (father's mother)
**CZE_10**	0	27	0	negative	0
**CZE_11**	0	58	0	**positive**	UtC (mother), GaC (father)
**CZE_12**	0	64	0	**positive**	HCC (sister)
**CZE_13**	0	NA	0	negative	0
**CZE_14**	0	18	0	negative	0
**CZE_15**	0	NA	0	negative	0
**CZE_16**	0	60	0	**positive**	PaC (father), BC (father's sister), RCC (mother's brother@60; mother's mother), LuC&HCC (mother's father)
**CZE_17**	0	44	0	**positive**	CRC (mother's father@65)
**CZE_18**	0	NA	0	**positive**	PaC (sister), BC (mother), GaC (mother's sister), OvC (mother's mother)
**CZE_19**	0	59	**BC**	**positive**	GaC (sister@50)
**CZE_20**	0	NA	**CRC**	NA	NA

BC - breast cancer; EC – endometrial cancer; GaC – gastric cancer; HCC – hepatocellular carcinoma; LuC – lung cancer; OvC – ovarian cancer; PaC – pancreatic cancer; PrC – prostate cancer; RCC - renal cell carcinoma; UtC – uterine cancer; @ - diagnosis at age

Figure S1: Survival of variant carriers in A. established genes, B. candidate genes, C. MRN genes compared to non-carriers.

A.

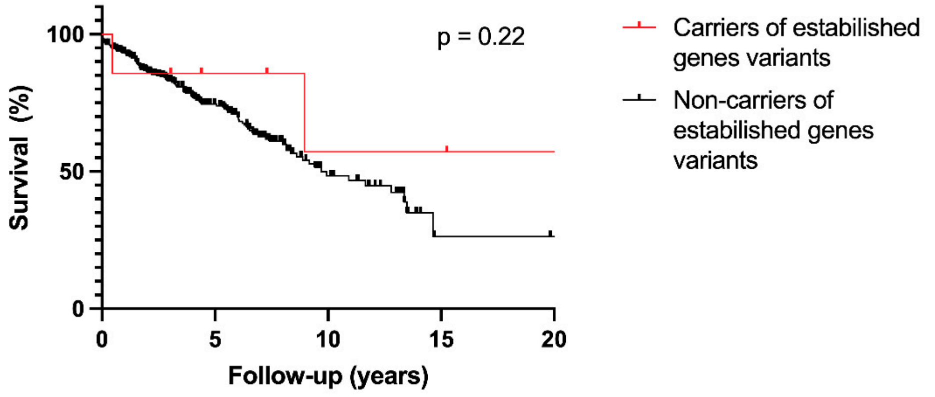

At risk					
Carriers (N)	7	5	3	2	1
Non-carriers (N)	327	106	33	3	2

B.

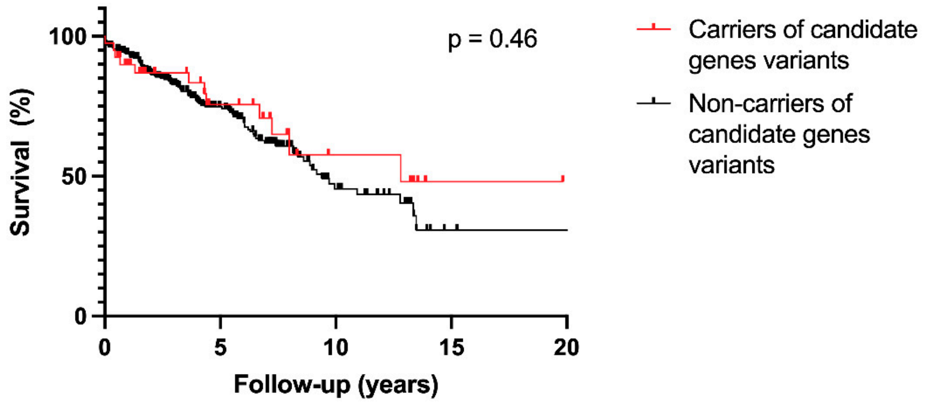

At risk					
Carriers (N)	40	19	7	2	1
Non-carriers (N)	294	112	26	3	2

C.

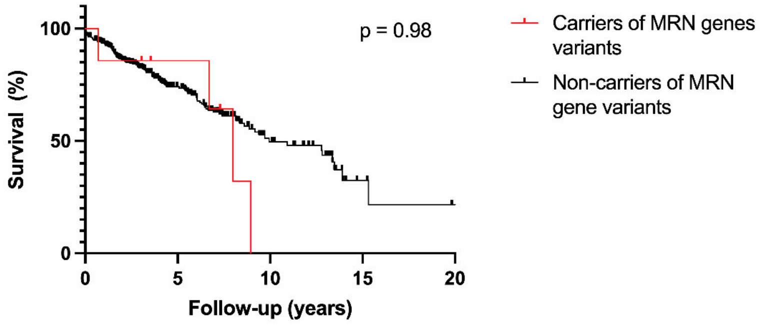

At risk					
Carriers (N)	7	5	0	0	0
Non-carriers (N)	327	127	34	5	2
